# Factors influencing choice of skilled birth attendance at ANC: evidence from the Kenya demographic health survey

**DOI:** 10.1186/s12884-018-1727-z

**Published:** 2018-04-10

**Authors:** Caroline Nyongesa, Xiaoyue Xu, John J. Hall, William M. Macharia, Faith Yego, Brigid Hall

**Affiliations:** 1 Centre for Clinical Epidemiology and Biostatistics, Hunter Medical Research Institute, School of Medicine and Public Health, the University of Newcastle, New Lambton Heights, NSW 2305 Australia; 2 Malteser International, P.O. Box 66587, Nairobi, 00800 Kenya; 3 Aga Khan University Hospital, 3rd Parklands Avenue, P.O Box 30270, Nairobi, 00100 Kenya; 4 Department of Health Policy and Management, Moi University, Nandi Road, Eldoret, 30100 Kenya; 5 Faculty of Medicine, UNSW Medicine, Wallace Wurth Building, The University of New South Wales, Sydney, 2052 Australia

**Keywords:** Maternal health, Skilled attendance, Demographic health survey, Kenya service provision assessment, Kenya

## Abstract

**Background:**

In Kenya, skilled attendance at delivery is well below the international target of 90% and the maternal mortality ratio is high at 362 (CI 254–471) per 100,000 live births despite various interventions. The preventative role of skilled attendance at delivery makes it a benchmark indicator for safe motherhood.

**Methods:**

Maternal health data from the Service Provision Assessment Survey, a subset of the 2010 Kenya Demographic Health Survey was analyzed. Logistic regression models were employed using likelihood ratio test to explore association between choice of skilled attendance and predictor variables.

**Results:**

Overall, 94.8% of women are likely to seek skilled attendance at delivery. Cost, education level, number of antenatal visits and sex of provider were strongly associated with client’s intention to deliver with a skilled birth attendant at delivery. Women who reported having enough money set aside for delivery were 4.34 (*p* < 0.002, 95% CI: 1.73; 10.87) times more likely to seek skilled attendance. Those with primary education and above were 6.6 times more likely to seek skilled attendance than those with no formal education (*p* < 0.001, 95% CI: 3.66; 11.95). Women with four or more antenatal visits were 5.95 (*p* < 0.018, 95% CI: 1.35; 26.18) times more likely to seek skilled attendance. Compared to men, female providers impacted more on the client’s plan (OR = 2.02 (*p* < 0.014, 95% CI: 1.35; 3.53).

**Conclusion:**

Interventions aimed at improving skilled attendance at delivery should include promotion of formal education of women and financial preparation for delivery. Whenever circumstances permit, women should be allowed to choose gender of preferred professional attendant at delivery.

## Background

Globally, almost 800 maternal deaths are reported each day in low and middle income countries. The increased lifetime risk of dying prematurely from pregnancy is one in 41 in developing countries, compared to one in 3300 in developed countries, making maternal mortality a social equity indicator [1, 2]. Maternal morbidity from serious injury or disability due to obstetric complications is also significantly higher in developing nations [2–4]. Maternal Mortality Ratio (MMR) is highest in Sub-Saharan Africa at 546 deaths per 100,000 live births [2] . This MMR is below the 75% reduction required to achieve the Millennium Development Goal (MDG) five targets, and far below the Sustainable Development Goal (SDG) 3.1 target of less than 70 deaths per 100,000 live births [2, 5, 6].

Kenya’s economic performance has seen it transform from a low to middle income country and despite the introduction of free maternal health services in June 2013 [7, 8], recent WHO trends (1990–2015) have placed Kenya among the top ten countries with the highest maternal mortality [2]. According to the 2014 Kenya Demographic Health Survey (DHS), although 96% of pregnant women received antenatal care (ANC) a significant proportion of women did not deliver at health facilities. The survey found an MMR of 362 (CI 254–471) [9] compared to the 2008/2009 DHS which found a higher MMR of 488 (CI 343–696) per 100,000 live births [10, 11]. As such, the MDG five target remains off track [5, 6, 11].

According to the World Health Organization (WHO), 80% of maternal deaths are preventable, even in low-income countries [3, 12]. Skilled attendance at delivery has been shown to be a surrogate marker for maternal mortality as approximately 16–33% of maternal deaths can be averted if supervised by a skilled professional [4, 11, 13–15]. The proportion of births attended by skilled health personnel is thus a benchmark used to monitor progress towards the achievement of MDG five and maternal health in the post-2015 era [6, 11, 13, 16]. Since service utilization is greatly determined by the perceived quality of care, it is crucial to comprehend the women’s perception of antenatal care (ANC) as an entry point into the continuum of maternal care and their level of satisfaction with the services provided [17]. Several studies have examined factors that promote or hinder use of maternal health services. However, these studies assessed factors within specific age groups, in specific regions, quality of health care and mainly within public health institutions [18–28]. Little is known of the demand for skilled birth attendance and which factors influence the choice of place of delivery. The choice of place of delivery affects if one receives skilled attendance at delivery, which in turn impacts maternal mortality. This study examined factors that influence the choice of skilled attendance at ANC.

## Methods

### Study design

We analyzed data from the 2010 Kenya Service Provision Assessment (KSPA) that forms part of DHS [29]. The KSPA surveys have been conducted every five years since 1999. In the 2010 KSPA, a nationally representative sample of 703 health facilities comprising of hospitals, health centres, maternities and nursing homes, dispensaries, clinics, and voluntary counseling and testing (VCT) centres were surveyed. All the facilities were randomly selected from Master Facility List (MFL) of 6192 operational facilities with the exception of three national referral hospitals and eight provincial hospitals. The KSPA survey provides information on essential health services such as child, maternal, family planning and reproductive health services [30]. The study used primary data from facility audit questionnaires termed facility inventories, and antenatal client exit interviews. This data was first cleaned and then analysed. A total of 1445 ANC client exit interviews, mainly from women of reproductive age (15–49 years old), were conducted. We excluded stand-alone VCT facilities as these were included only for information on HIV counselling and testing. Detailed descriptions of the sampling techniques and procedures applied for data collection are available in the KSPA [30].

### Outcome variables

The primary outcome variable was skilled attendance at delivery, defined as a delivery attended by a “health professional – such as a midwife, doctor, clinical officer or nurse” [29].

ANC attendants who indicated that they intend to deliver at a health facility, either current one or another were classified as likely to seek skilled attendance at delivery. Those who indicated that they intend to be delivered by either a Traditional Birth Attendant (TBA) or at a private home were classified as unlikely to seek skilled attendance at delivery. The Theory of Reason Action (TRA) by Ajzen and Fishbein (1980) [31] and Theory of Planned Behaviour (TPB) [32] state that a person’s behaviour is determined by their intention to engage in that behaviour and the perceived control over that behaviour. This is supported by a study in Nepal where intention to deliver in a health-care facility was associated with actual skilled birth attendance at birth as measured by birth preparedness [33].

### Predictor variables

The predictor variables were determined using the KSPA classification. Client satisfaction was based on the client’s overall opinion of ANC services and the duration clients waited before being attended. This opinion on quality of services was grouped under either “very satisfied”, “more or less satisfied” or “not satisfied” with the services given based on the response to what best describes your opinion of the services given today at the facility. Client’s response to whether or not they had money set aside for delivery was classified as no (client did not have any money set aside for delivery); yes, enough (client had enough money set aside for delivery) or yes, but not enough (client did not have enough money set aside for delivery). Other predictor factors that were considered based on significance in the literature and availability in KSPA data include service waiting time, age of client, highest level of education attended, total number of ANC visits, sex of the provider and region of residence [11, 18–22, 24, 34]. Waiting time was categorized into less than 30 min, 30 to 60 min, 61 to 120 min, and above 121 min. ANC visits were categorized into one, two to three, and four and above. Education level was ordered into no education and primary and above. Regions were grouped into two; Eastern and Northeastern representing the Arid and Semi-Arid Lands (ASAL) and Nairobi, Central, Western, Nyanza and Rift Valley, representing the non-ASAL.

### Statistical analysis

Data were analyzed using Stata 14.0 statistical software package (STATA, StataCorp, USA). Univariate and multivariate logistic regression analysis were used to explore the association between the client’s choice of skilled birth attendance and service cost and client’s over all opinion of the service, service waiting time, respondent’s age and education levels, total number of ANC visits, sex of service provider and geographical region of residence. The multivariable analysis used employed the Likelihood Ratio test, initially including variables in the model. The variables reporting *p*-values less than 0.05 on the Likelihood Ratio test were retained. The remaining variables were combined in one model and computed. Odds ratios (ORs), 95% confidence intervals and p-values are reported for univariate and multivariate analysis.

## Results

Of the 1445 participants, 99.2% (1397) were aged between 15 and 49 years. Characteristics of study participants are shown (Table 1). More than 90% had achieved primary level of education and above. Only 14% of the women had four or more ANC visits. 94.8% of women are likely to seek skilled attendance at delivery compared to 5% who are unlikely to seek skilled attendance at delivery.

**Table 1:** Characteristics of study participants

Total	n 1445	%	*P*-value
Age Median (IQR) 24 (21–29)			
Education level^✶^			< 0.001
No education	122	8.7	
Primary and above	1283	91.3	
ANC Visits^✶^			0.001
1	554	39.3	
2–3	656	46.6	
4 or Above	199	14.1	
Sex of Provider^✶^			< 0.001
Male	311	21.5	
Female	1134	78.5	
Regions^✶^			< 0.001
Non- ASAL	1168	80.8	
ASAL (Eastern & Northeastern)	277	19.7	

**✶**Significant differences have been found between education level, ANC visits, sex of provider and region (*p* < 0.001)

In (Table 2), the logistic regression models for skilled delivery, classified by predictor factor shows cost, education level, ANC visits and sex of provider are strongly associated with client’s intention to deliver with a skilled birth attendant at delivery. In particular, women who reported having enough money set aside for delivery were 4.34 (*p* < 0.002, 95% CI: 1.73; 10.87) times more likely to seek skilled attendance at delivery. Those with primary education and above had the highest odds ratio, which was 6.6 times higher than no education (*p* < 0.001, 95% CI: 3.66; 11.95). Women with four or more ANC visits were 5.95 (*p* < 0.018, 95% CI: 1.35; 26.18) times more likely to seek skilled attendance at delivery. The difference in odds ratios between male and female providers was 2.02 (*p* < 0.014, 95% CI: 1.35; 3.53) in favour of females.

**Table 2:** Logistic regression models for skilled delivery, classified by predictor factor

	n	Univariate model	Final Multivariate model✶		
	1445	Odds Ratio (95% CI)	*P*-value	Odds Ratio (95% CI)	*P*-value
Cost (has money for delivery)					
No	400	Reference			
Yes, enough	457	8.99 (3.76; 21.44)	< 0.001	4.34.(1.73; 10.87)	0.002
Yes, but not enough	552	2.80 (1.64; 4.76)	< 0.001	1.90(1.07; 3.35)	0.027
Opinion of ANC service					
Very satisfied	1161	Reference			
Not satisfied	223	0.89 (0.47; 1.68)	0.711		
more or less satisfied	22	0.97 (0.13; 7.40)	0.977		
Service Waiting time					
< 30	751	Reference			
30–60 min	255	1.55 (0.77; 3.12)	0.224		
61–120 min	170	1.32 (0.60; 2.85)	0.482		
> 121 min	233	2.40 (1.01; 5.70)	0.048		
Age of client					
1 year	1401	1.00(0.95; 1.04)	0.69		
Mother’s Education level					
No education	122	Reference			
Primary and above	1283	11.20 (6.55; 19.13)	< 0.001	6.62 (3.66; 11.95)	< 0.001
Total number ANC Visits					
1	554	Reference			
2–3	656	1.71 (1.04; 2.82)	0.04	1.58 (0.92; 2.7)	0.095
4 or more	199	7.95 (1.90; 33.24)	0.01	5.95 (1.35; 26.18)	0.018
Sex of Provider					
Male	311	Reference			
Female	1134	2.57(1.55; 4.25)	0.00	2.02(1.35; 3.53)	0.014
Geographical Region					
Non- ASAL	1168	Reference			
ASAL	277	0.31 (0.19; 0.52)	< 0.001		

✶ Odds ratio for final model adjusted for cost, education, ANC visits and sex of provider

## Discussion

Our study shows that majority of women (94. 8%) had planned to deliver through a skilled attendant. Our results also found other significant differences in women’s intention to seek skilled delivery in relation to cost (those having enough money for delivery having higher odds ratio than those with no money), education (primary education and above having higher odds ratio), ANC visits (four or more ANC visits had higher odds ratio) and sex of provider (with female providers having higher odds ratio than males).

According to the DHS 2008/9 data, only 43% of women were attended to by a skilled professional at delivery [11]. In Kenya, poverty, culture, and distance to health facility have been identified as factors that hinder women from utilizing skilled attendance at delivery. As our study focused on ANC attendees intentions, it is likely that many women in Kenya actually desire to be assisted by a skilled professional but they are constrained by these factors [10, 35]. It is also possible that some women may have responded to the KSPA planning for skilled attendance due to the perceived social desirability of this answer by the community [36].

Previous research has demonstrated a relationship between cost and access to maternal health care [10–12, 15]. The DHS data has shown that the proportion of children born at home decreased as mothers wealth increased. Our findings are consistent with these results [10–12, 15], whereby women who reported having enough money set aside for delivery were almost three times more likely to seek skilled delivery (*p* < 0.022) than those who reported having no money set aside for delivery. To address this barrier the government of Kenya introduced free maternal services in 2013. The impact of this policy on skilled attendance at delivery will require further analysis. Evidence on the implementation of the free maternal policy from Malaysia and Sri Lanka, who have achieved the MDG targets, demonstrated the importance of sustained national commitment as a key component to their success [37].

Education is a significant predictor of skilled attendance at delivery as shown by the differences between women with no education and those with primary and above. Educated women were almost 7 (*p* < 0.001, 95% CI: 3.66, 11.95) times more likely to opt for skilled attendance at delivery than those with no formal education. Previous studies and the DHS have also highlighted this finding [11, 18, 28, 38]. This finding points to importance of the role of education in the prevention of maternal mortality and morbidity. In order to achieve SDG 3.1, strong collaboration between the health and education sector is needed to ensure the achievement of SDG 4.1 which focuses on the education of girls [16, 39].

Our study revealed that women with four or more ANC visits were almost 6 times (OR 5.95, 95% CI: 1.35, 26.18) more likely to seek skilled delivery than those with only one visit. This link between ANC attendance and skilled delivery is similar to those in previous studies conducted in developing countries such as Kenya, Ghana and India [18, 26, 38, 40]. This supports the notion that antenatal care is a key pillar for safe motherhood because it offers an entry point into skilled care and the range of essential services that support the health of the mother and baby [26].

Interestingly, we found sex of the health provider at ANC to significantly influence intention to deliver with a skilled birth attendant at delivery. In particular, women were twice as likely (*p* < 0.014, 95% CI: 1.35: 3.53) to seek skilled attendance if attended to by a female provider than a male provider. Studies on gender preference have primarily focused on the doctor patient relations in obstetrics and gynaecology [41]. A few studies focusing on the ANC experiences of women in Cuba, Saudi Arabia and Thailand found that female doctors were preferred by women because they were viewed to be easier to relate with on an emotional level and also more likely to provide gender-related preventive services [41]. Very little data is available from developing countries, which are highly patriarchal and where gender concordance is necessary due to cultural and social practices that restrict physical contact between the sexes [41]. Further research is required in this area.

Overall, our findings reflect those highlighted in the strategic framework for the Elimination of Preventable Maternal Mortality (EPMM) [42]. In line with the framework, we support the need for a grand convergence of health and health enhancing sectors such as, education, social services and those focusing on gender empowerment for the achievement of SDG 3.1 of less than 140 maternal deaths per 100,000 live births in Kenya [2, 42].

### Limitations of the research

Several limitations should be considered. The cross-sectional study design employed by the KSPA means that causality cannot be established. This study was limited to only the facility audit questionnaires and ANC client exit interview, therefore the results cannot be extended to women who do not attend ANC. Certain demographic factors such as timing of first ANC, distance to health facility socio-economic status and urban/rural residence were not evaluated as they were not captured during the survey. Although traffic condition should be considered as a factor of the choice of skilled birth attendance, the KSPA dataset does not include such a question for analysis. Since the study utilized a secondary dataset certain variables were statistically insignificant possible due to low study power (service opinion, waiting time, age and region). We recommend that future KSPA surveys include a broader range of socio-demographic data in the ANC client exit interviews and consider the use of a similar unique identifier with the DHS to enable merging of service provision and household data.

## Conclusion

It is essential that programs aimed at improving skilled attendance at delivery as a surrogate marker for maternal mortality consider cultural factors such as the provider gender preferences of their clients. National interventions and policies need to focus on specific equity gaps relating to maternal education and the economic empowerment of women. As stipulated by the new Global Strategy for Women’s, Children’s and adolescent’s health and the SDGs, with regards to the reduction of maternal and child mortality, close attention needs to be given to the role of health enhancing and enabling sectors such as education [16, 39]. Due to the existing inequities in coverage, programs geared towards social protection and empowerment of economically disadvantaged women are necessary for the achievement of the post-2015 targets [16].

## Abbreviations

ANC: Antenatal care
ASAL: Arid and Semi-Arid Lands
CI: Confidence Interval
DHS: Demographic Health Survey
EPMM: Elimination of Preventable Maternal Mortality
KSPA: Kenya Service Provision Assessment
MDG: Millennium Development Goal
MFL: Master Facility List
MMR: Maternal Mortality Ratio
OR: Odds Ratio
SDG: Sustainable Development Goal
SSA: Sub-Saharan Africa
